# Supplementary material for: A set of multi-entry identification keys to African frugivorous flies (Diptera, Tephritidae)
Source: Zookeys. 2014 Jul 24;(428):97–108. doi: 10.3897/zookeys.428.7366 (PMC4143993; doi:10.3897/zookeys.428.7366)
Supplement: Supplementary material 6 — Key to Ceratitis [file zookeys-428-097-s006.zip › SF6_ZooKeys_key to Ceratitis/key/SF6_ZooKeys_key to Ceratitis/Media/Html/Ceratitis munroi.htm]

Ceratitis (Pardalaspis) munroi sp


***Ceratitis*** **(*Pardalaspis*)
*munroi* De Meyer**

*Ceratitis* (*Pardalaspis*)
*munroi* De Meyer, 1996 : 22.

Body
length: 7.0 mm; wing length: 7.0 mm.

Female

Head.
Antennal segments orange. Third antennal segment twice as long as second
segment. Arista basal part yellowish, otherwise dark. Frons pale  yellowish in
ground color, weak silvery shine in lower part; with dispersed short hairs,
distinctly darker than frons. Ocellar triangle dark. Face pale yellow. Occiput
moderately swollen below, pale whitish. Chaetotaxy normal for subgenus.

Thorax.
Ground colour of mesonotum beige with conspicuous orange tinge; longitudinal
lines hardly discernible. Postpronotum slightly paler than mesonotum.
Chaetotaxy normal for subgenus. Mesonotum with pale pilosity. Anepisternum
along upper margin and lower half with darker hairs. Two anepisternal
bristles.  Scutellum yellow with apical markings black; basally without dark
spots, only slightly darker coloration.

Legs
yellow-orange, setation typical of subgenus. Setae mainly dark, front femur
with posterior row dark. 

Wings
with yellow-brown bands. Banding, setation and venation normal for subgenus.
Marginal band continuous; discal band joined with marginal band; cross-vein r-m
at middle of discal cell; vein R_1_ ending before cross-vein r-m.

Abdomen.
Pale with orange tinge, with clearly defined but small spots. Pattern of spots
and setation normal for subgenus.  Oviscape shorter than abdominal terga 3-6,
yellow in colour

Male

Unknown.
